# “Scaling-out” evidence-based interventions to new populations or new health care delivery systems

**DOI:** 10.1186/s13012-017-0640-6

**Published:** 2017-09-06

**Authors:** Gregory A. Aarons, Marisa Sklar, Brian Mustanski, Nanette Benbow, C. Hendricks Brown

**Affiliations:** 1 Department of Psychiatry, University of California, San Diego, La Jolla, CA USA; 2 Child and Adolescent Services Research Center, San Diego, CA USA; 3 Department of Psychiatry and Human Behavior, Brown University, Box G-A1, Providence, RI USA; 4 Feinberg School of Medicine, Northwestern University, Chicago, IL USA

**Keywords:** Scaling-out, Scaling-up, Delivery system fixed, Population fixed, Implementation science, Evidence-based intervention, Intervention adaptation, External validity, Multilevel mediation modeling, Effectiveness, Mediational equivalence

## Abstract

**Background:**

Implementing treatments and interventions with demonstrated effectiveness is critical for improving patient health outcomes at a reduced cost. When an evidence-based intervention (EBI) is implemented with fidelity in a setting that is very similar to the setting wherein it was previously found to be effective, it is reasonable to anticipate similar benefits of that EBI. However, one goal of implementation science is to expand the use of EBIs as broadly as is feasible and appropriate in order to foster the greatest public health impact. When implementing an EBI in a novel setting, or targeting novel populations, one must consider whether there is sufficient justification that the EBI would have similar benefits to those found in earlier trials.

**Discussion:**

In this paper, we introduce a new concept for implementation called “scaling-out” when EBIs are adapted either to new populations or new delivery systems, or both. Using existing external validity theories and multilevel mediation modeling, we provide a logical framework for determining what new empirical evidence is required for an intervention to retain its evidence-based standard in this new context. The motivating questions are whether scale-out can reasonably be expected to produce population-level effectiveness as found in previous studies, and what additional empirical evaluations would be necessary to test for this short of an entirely new effectiveness trial. We present evaluation options for assessing whether scaling-out results in the ultimate health outcome of interest.

**Conclusion:**

In scaling to health or service delivery systems or population/community contexts that are different from the setting where the EBI was originally tested, there are situations where a shorter timeframe of translation is possible. We argue that implementation of an EBI in a moderately different setting or with a different population can sometimes “borrow strength” from evidence of impact in a prior effectiveness trial. The collection of additional empirical data is deemed necessary by the nature and degree of adaptations to the EBI and the context. Our argument in this paper is conceptual, and we propose formal empirical tests of mediational equivalence in a follow-up paper.

## Introduction

One goal of implementation science is to expand the use of evidence-based interventions (EBIs) appropriately and as broadly as feasible in order to foster the greatest public health impact [1]. This goal of generalizing the use of EBIs to improve public health is rooted in theory regarding external validity, first introduced approximately 60 years ago by Campbell [2]. In Campbell’s definition, external validity concerns the representativeness or generalizability of an effect and asks: to what populations, settings, and outcomes can an empirically established causal association between an intervention and outcome be generalized? Following this original formulation, Cook and Campbell [3] and Cronbach [4] specified domains wherein a causal association can be examined with respect to generalizability: the population where it is delivered, the intervention, outcomes, and settings. In this paper, we introduce a new concept for implementation called “scaling-out” where EBIs are implemented with either new populations, new delivery systems, or both. Using existing external validity theories and multilevel mediation modeling, we provide a logical framework for determining what new empirical evidence is required for an intervention to retain its evidence-based standard in this new setting. The goal of this paper is to present a conceptual approach to evaluating the spread and generalization of EBIs across health and allied health service systems within these domains.

When an EBI is implemented with fidelity in a setting that is identical to or very similar to where it was previously tested and found to be effective, it is reasonable to anticipate that the EBI would provide similar benefits to those found earlier. However, every EBI implementation raises two critical questions: (1) is there sufficient empirical evidence or justification from prior evidence that this EBI would impact health as expected, and (2) whether system, organization, and/or EBI adaptations are necessary, sufficient, and culturally and organizationally appropriate to make it feasible, practical, and acceptable in the new context. We argue first that EBI implementation in a moderately different setting or with a different population can sometimes “borrow strength” from evidence of impact in a prior effectiveness trial with additional empirical data deemed necessary by the nature and degree of adaptations. This strategy of testing precise elements in a mediation model can be seen as an extension of Cook’s five pragmatic principles for justifying generalized causal inferences to different target populations and settings [5]. We argue that adaptations to populations or delivery systems require that some new empirical evidence is often necessary to retain evidentiary status, and we lean on mediation modeling to make this case. While our argument in this paper is conceptual, we propose formal statistical approaches and empirical tests of mediational equivalence in a follow-up methods paper.

## Defining a new concept for implementation—“scaling-out”

We define the approach to adapting and delivering EBIs across health and allied health service systems and organizations and/or across different target populations as scaling-out. Scaling-out is the deliberate use of strategies to implement, test, improve, and sustain EBIs as they are delivered in novel circumstances distinct from, but closely related to, previous implementations. Although we propose an approach that identifies three types of scaling-out, we focus on two major types of scaling-out in this paper, (1) one that involves delivery of an intervention to the same target population as previously tested, but through different settings or delivery systems, and (2) one that involves delivering an intervention to a different population than previously tested, but through similar settings or delivery systems. The motivating questions are whether scaling-out can reasonably be expected to produce population-level effectiveness as found in previous studies, and what additional empirical evaluations would be necessary to test for this short of an entirely new effectiveness trial. If testing of this new scale-out requires the full empirical evaluation that would be required for establishing an EBI, this would be exceptionally costly, time consuming, and would delay implementation, especially to populations underrepresented in scientific trials or in settings where its delivery could reasonably produce benefit. Indeed, if we can legitimately borrow strength from previous studies and a modest amount of empirical evidence, this could accelerate and expand benefit to populations that have experienced health disparities that might never be included in a rigorous randomized effectiveness trial [6].

### Scaling-out vs scaling-up

The words “scaling,” “scale-up,” or “scaling-up” have clear meaning and importance in implementation science. In scaling-up, an EBI designed for one setting (e.g., a public mental health clinic) is expanded to other health delivery units within the same or very similar settings under which it has been tested (e.g., a statewide roll-out to all its public mental health clinics). An expectation of beneficial impact when scaling-up relies upon Cook’s principle of proximal similarity [5] because a nearly identical intervention is delivered in the same way to a similar population. Often when scaling-up an EBI to a large number of subjects by an expanded number of service delivery organizations, policy-makers and researchers are willing to assume that health outcomes will be improved as long as the EBI is implemented well [7]. Often, funders rely on this assumption without providing support for continuing evaluation for verification of impact. This minimalist perspective places a heavy reliance on previous tests of effectiveness and minimizes the importance of evaluations of implementation outcomes (e.g., self-reports of high fidelity). However, this perspective is not universally shared [8], and there are examples of EBI scale-ups where health outcomes did not improve as intended [9]. In some settings, policy-makers and system leaders want to know: will it (the EBI) work here for our citizens? Provided sufficient qualitative and quantitative data are available, modern implementation science evaluations can often be used to assess why expected implementation outcomes did or did not occur [10].

In contrast to this perspective on scaling-up, we use the term scaling-out to refer to specific variants in implementing an EBI, policy, or set of programs that are evidence-based. As shown in Table 1, we propose three types of scaling-out; the first variant, type I: population fixed, different delivery system, involves targeting the same population as previously tested, but through a different delivery system, the second type of scaling-out, type II: delivery system fixed, different population, involves targeting a different population than previously tested, but through the same delivery system, and the final type of scaling-out, type III: different population and delivery system, involves targeting a different population, through a different delivery system, as compared to the original EBI trial. In all variants of scaling-out, there is more concern about the impact on effectiveness and health outcomes, as there is more uncertainty whether the empirically supported causal association between intervention and outcome found in previous studies will hold when adapted and tested under yet-unstudied conditions. As a result, with scaling-out we are unable to rely completely on findings of previous studies.

**Table 1:** Key terms and definitions for the scale-out of an evidence-based intervention (EBI)

Key term	Definition
Scale-up	The deliberate effort to broaden the delivery of an EBI with the intention of reaching larger numbers of a target audience. Often an EBI scale-up will target health delivery units within the same, or very similar settings, under which the EBI has already been tested.
Scale-out	A deliberate effort to broaden the delivery of an EBI. Scale-out is an extension of scale-up and uniquely refers to the deliberate use of strategies to implement, test, improve, and sustain an EBI as it is delivered to *new populations* and/or through *new delivery systems that differ from those in effectiveness trials*. There are three types of scale-out, each indicating the extent to which the EBI is delivered to new populations and/or through new delivery systems.
Type I scale-out: population fixed, different delivery system	A type of scaling-out wherein an EBI is scaled-out to the same population as previously tested, but through a different delivery system.
Type II scale-out: delivery system fixed, different population	A type of scaling-out wherein an EBI is scaled-out to a different target population through the same delivery system as previously tested.
Type III scale-out: different population and delivery system	A type of scaling-out wherein an EBI is scaled-out to a different target population, through a different delivery system, than previously tested.
Borrowing strength	Utilizing empirical evidence from a previous EBI effectiveness trial in combination with new evidence from a scale-out trial to test EBI effectiveness when moving it to a new population and/or through a new delivery system. Borrowing strength allows for a more limited evaluation, typically prioritizing implementation outcomes, that takes less time and expense to conduct than the original effectiveness trial.
Intervention adaptation	Modifications to an EBI to facilitate its feasible, practical, and acceptable implementation in new contexts.
External validity	The representativeness or generalizability of an effect.
Core elements	Prototypical and/or necessary activities or components of an EBI. When scaling-out an EBI to a new population and/or through a new delivery system, core elements of the EBI should be retained to ensure its effectiveness.

*EBI* evidence-based intervention

We present a logical argument regarding the degree of empirical evidence needed for scaling-out that extends beyond the trials that established the original evidence of impact. Though we introduce three types of scaling-out, we focus on types I and II scaling-out in this paper, wherein either the target population or delivery system remains fixed, respectively. We note that the logic behind borrowing strength from previous studies requires that we justify that key elements of the intervention still exist and are delivered with fidelity, that the delivery system retains critical components of the implementation strategy, and that broader ecological systems are still supportive of the delivery and sustainment of this intervention. We describe four levels of evidence and recommend that evidence of the effectiveness of scaling-out can be supported if we establish that mediational pathways have equivalent strength as they did in the original trials.

### Empirical evidence needed for scaling-out

In one sense, the concept of scaling-out of an EBI is analogous to off-label use of a pharmaceutical that has been approved for patients having a specific indication. For example, the US Food and Drug Administration’s (FDA) and other regulatory agencies’ limited approval of medications to be used for specific conditions is designed to give strong assurance of what patients and physicians can expect under these limited settings. Except for specified contraindications, it is not illegal to prescribe FDA-approved drugs outside these settings (i.e., off-label usage) or at different dosages than originally approved. However, no assurance is given for their effectiveness upon doing so; there are no assurances of their safety, or that iatrogenic effects will be absent. Similarly, EBIs that are tested for effectiveness in one setting have some assurance of impact when scaled-up to health units and subjects in very similar settings. But we would have less confidence about the potential health impact of an EBI when scaled-out to different settings or populations. Intuitively one would expect that the more similar the delivery system, the broader the contextual setting, the intervention, and the population, the more we should be able to rely on previous evidence, and the less new empirical evidence should be needed, to anticipate a successful scaling-out.

But exactly what is the degree of “borrowing strength” from previous research studies that we would want to rely on? When is it justified to expect the same or similar impact of an EBI in this new setting? What new effectiveness testing should be required when an EBI is moved to different setting or population? What similarities exist in the mechanisms of action in the scaling-out compared to that previously found? When is it legitimate from a causal inference perspective to combine previously collected effectiveness and mechanistic evaluation data with new evaluation data on the scaled-out version? When would an entirely new effectiveness trial be required for a scaling-out to re-establish the existing standard of evidence?

To answer these questions, we begin with Cook’s conceptual principles of proximal similarity and heterogeneous irrelevancies [5]. Proximal similarity points to the degree that the scale-out contexts are similar to previous studies. A high degree of similarity engenders greater confidence that the health impact in the new context would be similar to what was found in previous studies. Heterogeneous irrelevancies refer to the robustness, or invariance, of a causal association across substantively irrelevant conditions. As noted by Matt and colleagues [11], “The greater the range of substantive irrelevancies across which a causal association has been found to be robust, the more confident one can be that the causal association will hold under yet-unstudied conditions” (p. 524). We propose criteria regarding when to turn these qualitative comparisons into empirical tests.

In our view, one must either establish or be willing to accept the following similarities between scaling-out and previous research. First, even as the EBI is adapted to new settings or populations, it still must retain its core elements [12]. Second, the underlying mechanism of action regarding how core elements affect health outcomes remains the same, which relies on analyses of such mechanisms [13]. Third, there must be sufficient organizational or system support to deliver the intervention as intended to sufficient numbers of the target population. Logically, we have no justification to anticipate health impact in scale-out if we are unwilling to embrace these three fundamental premises either based on the strength of generalizability of existing evidence or new data. Because scaling-out involves changes in the delivery system and/or population, and typically requires relevant EBI adaptation and/or context adaptation, we propose that an explicit mediation model be tested for equivalence to previous ones that established the intervention’s evidence [14].

## Two types of scaling-out: population fixed, different system, and delivery system fixed, different population, scaling-out

Here we emphasize two distinct types of scaling-out, “Type I: population fixed, different delivery system” and “Type II: delivery system fixed, different population” that we argue have potential for retaining their evidence-based status provided they also satisfy what we call “mediational equivalence” (discussed in greater detail below). In the discussion, we contrast these with both a typical scaling-up, wherein both the population and delivery system remain fixed, and the more complex scale-out, type III, wherein both the population and delivery system are different than originally tested.

### Type I: population fixed, different delivery system scaling-out

We formally define *type I: population fixed, different delivery system scaling-out* as implementation where an EBI is delivered through a different delivery system to the same population where it has previously been tested. This type of scaling-out pursues an alternative avenue to reach its target population. As an example, a number of evidence-based parent training programs have been tested and found to be effective in universal, selective, and indicated prevention trials [15–17]. Most of these programs have been tested and delivered in schools [18], mental health, or social services systems [19] but may have greater reach through alternative delivery systems. The first of two examples type I scaling-out involves the delivery of the SafeCare® child maltreatment intervention using an “interagency collaborative team” implementation strategy across one large (i.e., population = 3.2 million) county in the US [20]. In scaling-out SafeCare, a new interagency “seed team” was formed from diverse stakeholders that became the source of knowledge, model expertise, and leadership that allowed this EBI to be delivered with fidelity and to be sustained over time. The second example involves the delivery of Familias Unidas, a parenting program for Hispanic families with young adolescents, which was originally designed to be housed in middle schools [21–23]. Familias Unidas has been shown to have its strongest effect with Hispanic families with poor parent-child communication [24, 25], and it is this population that could be engaged through a different system than the school. Under policy mechanisms such as the US Affordable Care Act’s expanded access to health care in the US [26], there is an opportunity for Familias Unidas and similar EBIs to be accessible free of charge to the same types of families through primary care settings [27, 28]. Thus, health care system changes may provide an important opportunity for type I scaling-out. Because few effectiveness trials of these programs exist in primary care, the evidence for the effectiveness of such parenting programs while being delivered through primary care is relatively limited [29]. If we could legitimately build on the large effectiveness trial knowledge that already exists regarding these parenting programs, we could accelerate the research that supports delivery of such programs through primary care.

Successful implementation of these parenting interventions within primary care would require a reorganization to screen and refer families as well as to integrate, co-locate, or establish formal agreements between service systems, agencies, or community-based organizations that can deliver such programs effectively. In addition, type I scale-out may require important changes in how the intervention is delivered. For example, the Familias Unidas training program has been delivered by facilitators in small parent group meetings as well as in the home with individual families. SafeCare is delivered in the home where parents can learn and practice skills in vivo. A parenting program that is initiated by primary care may need to replace such group and home delivery modalities. This may be facilitated through the support of more logistically efficient technologies and content, such as interactive content about parenting viewable through tablets in primary care waiting rooms and virtual groups, having mock home environments within the primary care setting, or use of technology for parents to practice in their homes with coaches to work with them remotely. These sessions would support parents in practicing skills related to child safety, health, and parent-child interactions.

A final example of type I scaling-out is the delivery of pre-exposure prophylaxis (PrEP) to populations at high risk of HIV infection. In international and domestic clinical trials, adherence to PrEP medication has been demonstrated to reduce the risk of HIV infection by close to 90% among adult sero-negative men and women whose partners were infected with HIV and at-risk adult men who have sex with men, populations for whom federal guidelines released by the Centers for Disease Control and Prevention (CDC) recommend use of PrEP [30]. In order to expand the use of PrEP, clinics serving these populations that conduct HIV/STD testing and treatment are emerging as sites for offering and/or delivering PrEP. However, PrEP delivery requires a higher level of engagement of healthcare providers than has typically been engaged for HIV primary prevention; in fact, it more closely mimics the types of care an individual living with HIV received than the less medicalized services more typically provided for HIV prevention. Staffing, training, and costs required to deliver PrEP in this setting are complex and need to be addressed for successful implementation in STD clinics [31].

### Type II: delivery system fixed, different population scaling-out

We define type II: delivery system fixed, different population scaling-out as implementation that extends the reach of an existing intervention to a novel population within a similar service system. Type II scaling-out uses the same delivery system but aims to reach a different population of individuals, groups, or families for which the intervention has not yet been tested. For example, a smoking cessation approach originally tested with behavioral health patients may be implemented for those with diabetes in the same managed care health system. However, populations may also vary by race or ethnicity, cultural heritage, and considerations would have to determine what core elements must be retained and what might be adapted.

A large literature exists on cultural adaptation of evidence-based interventions [32, 33]; many of these are examples of scaling-out, as often the delivery system is held constant. There already exist several approaches to surface and deep structure adaptation to different populations [34], and the framework in this paper can complement these approaches by identifying particular components to test empirically.

PrEP delivery for adolescents is an example of a type II: delivery system fixed, different population scaling-out implementation. The CDC PrEP guidelines [30] did not provide a recommendation for the use of PrEP for at-risk adolescents due to lack of evidence of efficacy and safety for this population at the time the guidelines were developed. However, adolescents between the ages of 13–19 who engage in risky sexual behavior and/or injection drugs are also at increased risk of HIV infection, and accounted for 4% of new HIV diagnoses in 2015. Recognizing this potential risk, health care providers have begun to deliver PrEP for adolescents. Given that state laws and regulations vary in terms of parental consent requirements for medical services, PrEP implementation in this new population requires different strategies and research that can inform effective delivery [35–37].

## Evaluation options for scaling-out

### EBI replicability or effectiveness

When scaling-out, having metrics to test specifically whether expected outcomes improve are important; however, we suggest that there are instances where it may not be necessary to include all, or even any of the health outcomes that might be included in an efficacy or effectiveness trial. To assess whether a scaled-out version has impact on the ultimate health outcome or distal target of interest, we propose an efficient evaluation that tests a limited set of means and relationships, and combines new data with evidence from previous trials. Typically, a more limited evaluation would collect a small amount of—or even no—health outcome data, but a substantial amount of implementation process and output data, what others have called implementation outcomes [10]. It would therefore take far less time and expense to conduct such an evaluation compared to the original effectiveness trial.

### Levels of evidence for scale-out evaluations

As shown in Table 2, we have identified four levels of evidence that could be applied to predict or measure the expected health impact of implementing an EBI. In this table, the columns refer to major domains of the RE-AIM model that have long been recognized as critical in producing population-level effects [38]. Contents of other columns provide examples of potential constructs of interest. The empirical data in these implementation studies range from no new data in level 0, to a complete replication trial that again supports EBI effectiveness in level 3. Level 0 depends almost exclusively on the assumption that the new implementation will follow in the same footsteps as the previous intervention did when it produced findings of health impact. With a lack of empirical data in the new setting, the burden of proof necessarily rests on similarity with previous work. Level 1 involves proxy or indirect measures of the key RE-AIM components and is intended as an inexpensive large-scale implementation evaluation. Level 2 focuses on demonstrating that key theoretical mediators or mechanisms work as expected. Finally, level 3 involves a full-scale randomized study such as a type 2 hybrid trial that tests both effectiveness and implementation [39]. We note that an actual design may involve different levels of assessment across the columns. For example, it may be appropriate to use a full randomized trial to assess a proxy outcome (e.g., level 3) while not measuring the ultimate or distal health outcome (level 0).

**Table 2:** Four levels of evidence for evaluations and examples in scaling-out an evidence-based intervention (EBI)

Level Of evidence	Implementation fidelity (Implementation strategy delivered as intended)	Intervention fidelity (Clinical or health intervention delivered as intended)	Reach and exposure	Adoption	Sustainment	Effect on health outcome	Potential use
0: minimal or no new empirical evidence	Not measured	Training certification of facilitator and/or clinician prior to new implementation	Numbers of individuals exposed	Attendance of organizational representatives at trainings	Not measured	Not measured	Demonstration program that explicitly follows an intervention manual
1. Proxy empirical evidence	Leadership and staff self-efficacy to support EBI	Facilitator and/or clinician ; self-assessment of fidelity	Attendance for behavioral intervention; filled prescriptions	Formal acknowledgment by organizations of adoption	Completion of yearly reports by implementing agencies	Assessment of intermediate and/or proximal health outcome	Inexpensive large-scale implementation evaluation
2. Direct empirical evidence	Measurement of milestone attainment; speed, quality, and quantity of implementation	Independent assessment of fidelity	Ratings of quality of behavioral homework, medication adherence	Quality of staff training	Sustained number of staff and number of subjects exposed to intervention with fidelity	Change in primary health outcome from baseline	Formal implementation evaluation to establish evidence base through mediational mechanisms
3. Full randomized hybrid trial						Evaluate intervention vs comparison on primary outcome	Type II hybrid trial to directly establish full evidence base

### Sequential mediating model for assessing EBI effectiveness

**Fig. 1:**
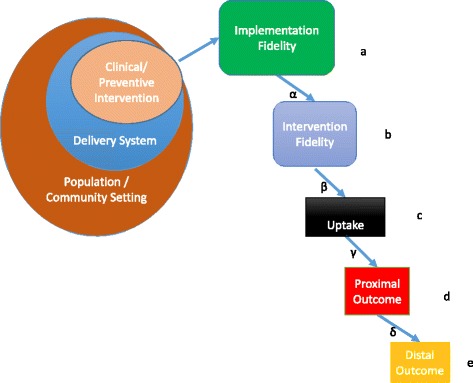
Schematic of scaling-out and implementation and effectiveness domains for evaluation

To examine when such a limited evaluation design for scaling-out would be sufficient to judge whether the EBI retains effectiveness, we consider a simplified sequential mediational model presented in Fig. 1. This figure provides a schematic view of the major domains to examine regarding whether a clinical/preventive intervention that has been judged to be evidence-based within one setting would be expected to have similar effects when scaled-out. On the left, a specific clinical/preventive intervention is embedded in a health delivery system and ecological context including characteristics of the population, local communities, and macro system [40]. In scaling-out, the intervention and/or context could be adapted and either the health delivery system/community context or population is different from that in which it was originally tested. At the top right of this figure are two factors central to health outcomes from a delivery perspective: fidelity of the implementation process known as “implementation fidelity” (e.g., training, supervision, incentive structure, and informatics) [41] and fidelity to the clinical/preventive intervention itself known as “intervention fidelity” (or more generally this includes adherence to the intended program content, and responsiveness, quality and competence in delivering the program [42, 43]). Next is the degree of uptake by the target population (e.g., the reach into the target community and the degree of exposure to, or usage of the clinical/preventive intervention). Following this in sequence are the proximal behavioral outcomes (e.g., changes in parent-child communication, medication adherence, and selection of evidence-based prevention programs that match community needs) and the ultimate health outcomes (e.g., reduced HIV infections for PrEP or parenting skills, children’s cognitive, affective, or behavioral health for parenting programs) [6]. The Greek letters in this figure indicate the strength of relationships between the steps in this mediational sequence, and the Roman letters represent mean levels achieved for each of these.

## Assuming a conceptual theory of mediation holds for scaling-out

Cook’s principle of causal explanation [5] requires investigation into the causal mediating mechanisms that underlie a relationship of interest, explaining how and why an effect occurs. Complete understanding of a causal mediating process can strengthen generalized causal inference by providing information on when and where an effect can be replicated. As one example, improvement in the parent-child relationship through parenting interventions has been shown to relate to lower drug abuse, HIV sex risk behavior, and internalizing behavioral symptoms for adolescents [24, 44–46]. Thus, the parent-child relationship can serve as a proximal mechanism or intermediate outcome on the pathway to improved behavioral health. In delivering PrEP to prevent HIV incidence, adherence to the medication would be the most important intermediate outcome. Assuming the conceptual theory of mediation holds in a scaled-out adaptation, relying on Cook’s principles of heterogeneous irrelevancies and proximal similarity, then we could assess impact on the mediating variable using a level 1 design for assessing a proxy outcome. We can also infer that positive effects would be expected to translate into corresponding benefit on the distal outcome as well. Following up the example of Familias Unidas interventions, Perrino and colleagues tested the impact of this intervention in an integrative synthesis analysis on three populations ranging from a general population (universal prevention) to one with moderate risk (selective prevention) and more serious individual level risk (indicated prevention) [24]. They found that the theoretical mediator of parent-child communication was impacted only when its baseline measure was low. Therefore, it would be reasonable to expect beneficial outcomes from this intervention when an intervention increases the mean on parent-child communication to the level found in previous studies.

## Considerations for scaling-out

### Intervention adaptation

For all types of scaling-out, it may be possible to use the identical EBI (e.g., delivered using the same manualized version and/or dosage). However, we more often need to adapt the intervention to fit the new population and/or new delivery system. To further describe the adaptations that need to occur during scaling-out, we build on commonalities that are present in three implementation frameworks, the Exploration, Preparation, Implementation, Sustainment (EPIS) framework [47], the Dynamic Sustainability Framework [40], and the Consolidated Framework for Implementation Research (CFIR) [48]. In addition, the EPIS framework led to the development of the Dynamic Adaptation Process (DAP) that provides a process for pre-assessment, convening an “implementation resource team” to guide the implementation process, and use of audit and feedback data to help guide appropriate EBI adaptation [49]. The important commonalities across these frameworks are that they all address outer policy and system context, inner organizational context where services are delivered, and characteristics of the EBI itself. Thus, for successful scaling-out an EBI, we focus on three key interconnected implementation/sustainment components: (1) the alignment of the surrounding ecological context including characteristics of the target population as well as broader cultural and contextual factors (e.g., policies, funding), (2) the alignment of the health or service delivery system and organizations where it is delivered, and (3) the integrity of the EBI itself as adaptations occur. These three components often evolve through external forces (e.g., national health system policy reform and/or reimbursement), change in response to interactions during scaling-out (e.g., a children’s hospital hires community outreach workers to deliver a new EBI in high-need communities), or change when an EBI is adapted to patients or clients (e.g., making an intervention acceptable to those with a different cultural background).

Concern regarding the effectiveness of an intervention when adapted has been addressed for a number of specific EBIs, including child maltreatment interventions [50], substance abuse treatment [51], child anxiety interventions [52], HIV treatment [53], school-based social competence interventions [54], psychological treatments for a variety of disorders [55, 56], and health risk prevention programs [57–59]. However, there is mounting evidence that overly strict intervention fidelity may be at odds with effective implementation of EBIs in real-world practice settings (i.e., outside of highly controlled efficacy trials), thus, raising concern about the balance between delivering EBIs with fidelity and making adaptations believed to be necessary for usual care contexts. This “adaptation-fidelity” tension is a critical component of scaling-out in that it is addressed head on as proposed in more dynamic models of implementation process [60–62]. However, new approaches to identifying and coding EBI modifications and adaptations are promising in regard to rigorous study of adaptations and their impacts [63]. Scaling-out, while allowing for appropriate system, organization, and intervention adaptation, necessitates a better understanding of how to facilitate delivery of EBIs with appropriate content adherence and competence in delivery, while allowing for adaptations to facilitate effective uptake and spread, and that do not interfere with core elements (i.e., intervention components believed to be necessary to attain intervention effects).

### Core elements of the intervention

One of the first steps in scaling-out is to delineate the core elements of the EBI to be implemented. We define core EBI elements as activities or components of an intervention that are necessary in order to obtain the clinical or public health outcomes [58, 64–66]. If core elements are well-defined then it is possible to determine what is and what is not adaptable [48] (at least for the EBI). To have maximum positive impact, any adaptations of an EBI to a new context should retain the core elements and add or modify components that complement, and do not conflict with existing ones. This concept of retaining core elements to facilitate generalization of impact is consistent with Cook’s (1991) principle of proximal similarity [5]. Under this principle, generalization is justified when all relevant properties of the causal association, such as prototypical and necessary components of an EBI, are adequately represented in the new context. Reciprocally, knowledge of irrelevant components of an EBI also facilitates decisions regarding adaptations to fit a new context. This is consistent with Cook’s principle of heterogeneous irrelevancies that suggests the greater the range of substantive irrelevancies across which a causal association has been found to be robust, the more confident one can be that the causal association can be generalized to yet-unstudied conditions. Discussions of this issue most commonly pertain to adaptations to fit an EBI for a particular cultural group [58]. However, adaptations to accommodate an EBI are likely to be needed at the service system or organization levels, and often to the EBI itself [47, 60]. The less system/organization adaptation needed, the more readily an EBI can be assimilated. However, systems and organizations may need to adapt or accommodate and change in order to implement and sustain a given EBI. In our framework, the existence of multiple trials that produce similar health outcomes, such as one finds in a synthesis of preventive trials focused on child depression [67], makes for a higher expectation of health impact when a scaled-out intervention shows comparable intervention and implementation fidelity and reach as in previous studies.

## Discussion

In this paper, we introduced and defined a new concept for implementation called scaling-out. Scaling-out provides an opportunity to use a strategic approach to improve the efficiency of moving an EBI from one setting to another and/or from one population to another. When scaling-out an EBI in a moderately different setting or with a different population, we suggest it is sometimes possible to “borrow strength” from evidence of impact in a prior effectiveness trial with additional empirical data deemed necessary by the nature and degree of adaptations. We take a mechanistic approach in suggesting that by testing underlying mechanisms or mediators of effects, the efficiency of testing EBIs for new populations or new service systems can be streamlined through a greater understanding of and use of prior data to borrow strength from previous effectiveness studies.

### Scaling-out

We have focused primarily on two types of scaling-out in this paper for a single EBI and recommended different levels of empirical tests involving key mediators that would provide some assurance that the scaled-out implementation would produce its intended effects. Similar scaling-out approaches are possible when implementing other types of interventions including evidence-based decision support systems, such as Communities that Care [68–72], which helps communities decide which among a menu of EBIs are most appropriate to implement, rather than a single intervention. We only mention the important and more complex type III scale-out, wherein both the delivery system and the population are different than in initial efficacy studies. For example, interventions to prevent mother to child HIV transmission (PMTCT) that are moved from clinic-based to congregation-based services in different countries or service systems could engage different populations that were not included in previous trials [73]. It seems natural to require much more evidence when both population and delivery system change; doing so would demand new empirical evidence beyond that for either delivery system or population fixed scaling-out.

The notion of level of similarity of a population or delivery system is an important consideration. Take type I scale-out first, wherein the population remains fixed and the delivery system is different. Type I scale-out will require implementers to develop ways in which population differences may be minimized. Inclusion/exclusion criteria for those to receive a given EBI could be utilized to clearly (as much as possible) define the service population. For example, adolescents with juvenile justice system involvement, a substance use disorder, and of a similar cultural background could be identified. For type II scale-out, wherein the delivery system is fixed, though the target population is different, there also could be multiple approaches. For example, San Diego County Mental Health and Los Angeles county Mental Health are both large public sector service systems operating within the same state in the US. They both provide services to a large extent through some direct service provision, but primarily provide services through procurement and contracting with community-based organizations to provide direct services to children and adolescents. While these systems do share some similarities, there are also some differences in regard to their procurement and contracting processes.

For different types of scaling-out, we propose a logical framework that researchers and policy-makers can use to assert conditions under which an established sequence of mediational elements described in Fig. 1 could (a) be expected to hold in the absence of new data, (b) be ascertained by new proxy or direct empirical evidence that their mean values are equivalent to that from previous studies (Roman letters in Fig. 1), or (c) be ascertained to retain the same mediational relationships (Greek letters in Fig. 1). Table 3, which presents a two-by-two table where population and delivery system are either fixed or different, summarizes levels that one would ordinarily be willing to assert are needed to retain evidentiary standards. Specifically, the upper left cell involves traditional scaling-up, covering the situation where the population and delivery system remain the same. Sequential mediational equivalence for scaling-up is often presumed to hold, so one would typically require low levels of evidence for the means (Roman letters; mostly levels 0 or 1), no need to reverify that the mediational relationships hold (Greek letters), and no need to measure distal outcomes. For type I population fixed, different delivery system scaling-out (upper right), the mediational relationships are again expected to hold, reach could be measured with a proxy, but because the delivery system changes we would need stronger evidence around implementation fidelity, adoption, and sustainment. For type II delivery system fixed, different population scaling-out (lower left), we would typically want to assess reach with high accuracy due to the focus on a new population, but may make the case that assessing implementation fidelity, adoption, and sustainment could be done with proxy measures. For type III scaling-out where both population and service delivery are different (bottom right), logic would require more empirical examination that the mediational pathways remain in this new context compared to types I or II scaling-out. Potentially, proxy measures could be used to assess these mediational relationships, and generally some measures of the distal health outcome would be required as well.

**Table 3:** Typical Levels of Evaluation Required when Population and/or Delivery System Change

			SYSTEM	
		Domain (mean, regression from Fig. 1)	Same	Different
POPULATION				
	Same		*Scaling-Up*	*Type I Scaling-Out: Population fixed, different delivery system*
		Implementation Fidelity (a, α) Intervention Fidelity (b, β) Reach (c, γ) Adoption (a, α) Sustainment (a, α) Health Outcome (d, e, δ)	a = 1–2, α = 0 b = 1–2, β = 0 c = 1, γ = 0 a = 1, α = 0 a = 1, α = 0 d, e = 0, δ = 0	a = 2, α = 0 b = 1–2, β = 0 c = 1, γ = 0 a = 2, α = 0 a = 2, α = 0 d, e = 0, δ = 0
	Different		*Type II Scaling-Out: Delivery System Fixed, different population*	*Type III Scaling-Out: Different Population and Delivery System*
		Implementation Fidelity (a, α) Intervention Fidelity (b, β) Reach (c, γ) Adoption (a, α) Sustainment (a, α) Health Outcome (d, e, δ)	a = 1,2, α = 0 b = 1–2, β = 0 c = 2, γ = 0 a = 1, α = 0 a = 1, α = 0 d, e = 0, δ = 0	a = 2, α = 1–2 b = 2, β = 1–2 c = 2, γ = 0 a = 1, α = 0 a = 1, α = 0 d, e = 2–3, δ = 0–3

Notes: Refer to Fig. 1 for definitions of Greek and Roman symbols

Turning such guidance on the levels of new knowledge required, as presented above, into a rigorous system of empirical evaluations for non-inferiority compared to previous findings, will require additional development and explication of statistical methods that will be presented in a follow-up paper. At the heart of the scale-out approach is borrowing strength through the use of mediational modeling. Mediational analysis has an extensive history [74, 75] but has had increased interest in implementation science [76]. Recent methodologic developments have addressed challenging issues in causality for single [77] and multiple randomized trials [25], evolving interventions [12], natural experiments [78, 79], and multilevel [78] as well as multidimensional situations [80].

This proposed framework for implementation research regarding scaling-out still leaves unanswered details regarding how best to assess changes in the complex implementation systems that we construct to deliver EBIs. Major implementation strategy adaptations often occur, and indeed are sometimes required to support implementation in delivery systems with widely different system and/or organizational cultures and climates, readiness, and resources [81], to populations having widely different histories, norms and values. Indeed, there is a recognition that equifinality [82] is common in complex systems governing implementation. That is, there are multiple implementation strategies that, in specific circumstances can effectively address different barriers to implementation, as well as the same barrier (e.g., financing) [83–85]. Nevertheless, it is likely that broad systems for measuring implementation process through key milestones, quality, and quantity, such as those identified in the Stages of Implementation Completion [86–88], or similar unobtrusive measures [89] can be used to measure implementation fidelity and progress in diverse conditions.

## Conclusion

To close this discussion of a framework for asserting that a scaling-out is expected to share the original version’s impact on health outcomes by relying on previous studies and new empirical data, we note that more rapid implementation is especially important to the delivery of effective interventions to minorities and other populations experiencing health or health service disparities that would otherwise not benefit from the extensive research required to demonstrate effectiveness [90, 91].
